# The impact of time to diagnosis on health service use, cost, and quality of life for patients with juvenile idiopathic arthritis: a cost-utility analysis

**DOI:** 10.1186/s12969-026-01195-7

**Published:** 2026-02-24

**Authors:** Amy Von Huben, Diana M. Bond, Samantha Lain, Davinder Singh-Grewal, Ruth Colagiuri, Stephen Colagiuri, Natasha Nassar

**Affiliations:** 1 Leeder Centre for Health Policy, Economics and Data, Faculty of Medicine and Health, The University of Sydney, Sydney, Australia; 2 Child Population and Translational Health Research, The Children’s Hospital at Westmead Clinical School, Faculty of Medicine and Health, The University of Sydney, Sydney, Australia; 3 Department of Rheumatology, The Sydney Children’s Hospitals Network, Sydney, Australia; 4 John Hunter Children’s Hospital, Newcastle, Australia; 5 Juvenile Arthritis Foundation Australia (JAFA), Sydney, Australia; 6 Boden Collaboration, Charles Perkins Centre, The University of Sydney, Sydney, Australia; 7 Charles Perkins Centre, The University of Sydney, Sydney, Australia

**Keywords:** Juvenile idiopathic arthritis, Economic evaluation, Health-related quality of life, Cost-utility analysis, Cost-benefit analysis

## Abstract

**Background:**

Timely referral and diagnosis of Juvenile Idiopathic Arthritis (JIA) by a pediatric rheumatologist ensures early intervention to minimize long-term joint damage and disability. This study aimed to quantify the impact of delays in diagnosis on health service use, health-related quality of life (HRQoL) and associated costs to the health system.

**Methods:**

A cost-utility analysis was conducted over a lifetime horizon from a health funder perspective in 2023 Australian dollars, comparing time from actively seeking treatment to formal diagnosis (< 6 months, 6 + months). Time to diagnosis, healthcare use, including medical investigations, health professional visits, hospitalizations and medications, was determined using The IMPACT Survey. Incremental costs were estimated by applying Australian government subsidy item costs (medications), schedule fees (medical services), and National Efficient Prices (hospitalizations). HRQoL was measured using Child Health Utility Instrument (CHU9D). Incremental Quality Adjusted Life Years (QALYs) were estimated using participants’ CHU9D utility values based on Australian preference weights and life expectancy. Costs and QALYs were present valued at 5% per annum. Sensitivity analyses were conducted for robustness.

**Results:**

Over one-third (*n* = 61/163) of children were diagnosed 6 + months from actively seeking treatment. Compared with 6 + months, diagnosis within 6 months was associated with a lower use of health services, resulting in a mean annual decrease in costs for the healthcare funder of more than $10,000 per child. The majority of the cost savings were due to reductions in hospitalizations for pain, inflammation, and investigative procedures. There were also significant increases in HRQoL; 0.14 (95%CI 0.05, 0.23) utility value. The differences in health service use and HRQoL from timely diagnosis persisted over 20 years from diagnosis. Over a lifetime, the present value of healthcare cost savings was $208,458 (95%CI $45,388, $371,528) and the increase in HRQoL resulted in an additional 2.82 (95%CI 1.03, 4.61) QALYs per child. At a willingness to pay of $50,000 per QALY, the estimated net benefit to the health funder was $349,520 (95%CI $139,630, $559,411) per child.

**Conclusions:**

Interventions to improve the time to diagnosis of JIA within six months are likely cost-effective and can significantly improve HRQoL for people living with JIA.

**Clinical trial registration (if any):**

Not applicable.

**Supplementary Information:**

The online version contains supplementary material available at 10.1186/s12969-026-01195-7.

## Background

Juvenile Idiopathic Arthritis (JIA) is an inflammatory arthritis of unknown cause and onset before 16 years of age that persists for at least six weeks and comprises 80% of all Childhood Rheumatic Diseases [1]. Although JIA incidence has been reported to range widely, 1.6–23 per 100,000 [2], recent population-based register studies using the International League of Associations for Rheumatology (ILAR) classification criteria have estimated annual incidence in the range of 24.1 [3]–31.7 [4] per 100,000; similar to childhood diabetes [5], yet there is less political, community, and health professional awareness of the impact of JIA on individuals, families, and health systems.

JIA is characterised by joint pain, swelling and inflammation, which, without timely referral to a Pediatric Rheumatologist (PR) for aggressive intervention with disease-modifying drugs, can lead to long-term joint damage and disability [6–9]. Timely diagnosis also aids appropriate management of co-morbidities such as uveitis, preventing vision loss [1]. Large longitudinal studies of health-related quality of life (HRQoL) in the era of biologic treatments for JIA have shown that a longer time to diagnosis predicts a poorer HRQoL trajectory over the first three years post-diagnosis [10], which is the median time to reach maximal HRQoL once on treatment [10, 11]. This early decrement in HRQoL continues into adulthood, where pain intensity, active joints, and physical disability within the first three years are early predictors of physical limitations, pain, and poorer physical HRQOL after 19 years [11]. Chronic pain, even for those in remission, most likely from radiological damage before treatment, reduces long-term HRQoL and increases long-term health system costs, particularly for joint replacement surgery [12], strengthening calls for earlier diagnosis. The findings highlight the importance of early diagnosis and treatment of JIA to prevent lifetime decrements in HRQoL and health system costs.

Despite clinical guidelines recommending that a child with suspected JIA should be assessed by a PR within 10 weeks (2.3 months) of symptom onset [13], a 2020 international systematic review found the pooled median time from symptom onset to first PR assessment was 5.8 (95% CI 5.3, 6.3) months [14]. The delay in diagnosis [14–16] of JIA and the drivers of this delay [1, 8, 14, 16, 17] have been well documented. However, to our knowledge no studies have quantified the incremental impact of the delay in diagnosis on a child’s health-related quality of life HRQoL (physical, psychological, and social functioning) and health system costs for use by policy makers and health funders. This quantification work is essential to provide impetus to fund interventions to improve time to diagnosis.

An online national survey, The IMPACT Survey [18], was undertaken among Australians with JIA and their families from February to June 2023, to capture the experience of diagnosis, treatment, healthcare access and interactions, and to measure HRQoL and the financial costs associated with treatment and care. The survey identified for the first time that HRQoL for Australian children with JIA was lower than that of children with epilepsy or Type 1 diabetes [18]. The survey also confirmed that health system costs associated with the condition were high, and the average time to diagnosis for JIA was 11 months [18], eight months longer than the recommended standard [13]. However, there was no assessment of whether the time to diagnosis had a significant impact on HRQoL and health system costs.

The aims of this study are twofold: (1) To quantify the relationship between time to diagnosis and its impact on HRQoL, Quality Adjusted Life Years (QALYs), and health system costs, and (2) Estimate the net benefit in dollars to healthcare funders of timely diagnosis. This study differs from a typical cost-utility analysis in that it does not evaluate a particular intervention for cost-effectiveness. Rather, the health economic modelling undertaken in this paper provides health funders and policy makers with an estimate of the dollar cost (QALYs forgone and health system costs) per person of a delayed JIA diagnosis, or alternatively, the threshold (or maximum) funds per person that can be spent on a particular intervention to improve time to diagnosis. The paper shows how the threshold, along with estimates of the children affected in the relevant country, can be used to set a budget for improving time to diagnosis. Finally, with the inclusion of the funds required per-person to implement a particular intervention, the cost-utility and cost-benefit of any intervention to improve time to diagnosis for children with JIA to within six months of actively seeking treatment can be evaluated with the estimates provided in this paper.

## Methods

### Study population

Australians aged up to 25 years with JIA diagnosed before age 16 and their families were recruited through consumer organisations and paediatric rheumatology services and completed the IMPACT Survey between February and June 2023. Detailed methods are published elsewhere [18]. Ethics approval was obtained from The University of Sydney Human Research Ethics Committee (HREC: 2022/902).

### Data collection

The IMPACT Survey was designed and piloted by a multidisciplinary team of researchers with expertise in child/adolescent health research, members of the Australian Paediatric Rheumatology Group, and people with lived experience of JIA. It was administered online via REDCap as a one-time, cross-sectional survey. The survey collected information on sociodemographic and clinical characteristics, process of diagnosis, healthcare interactions, treatment, physical and mental health, social/family impact, government and out-of-pocket costs and health-related quality of life. Parents/carers and children/young people (hereafter referred to as children/child) were encouraged to complete the survey together, and more than half the participants (54%) reported they completed the validated quality of life measures together, which did not differ by time to diagnosis groups (*p* = 0.3).

### Time to diagnosis measure

Respondents were asked to estimate the time from actively seeking treatment to formal diagnosis of JIA, which was captured in categories of < 1, 1–2, 3–5, 6–8, 9–12 months, and 1–2, 3–4, > 5 years for all participants. Respondents were also asked to report age at symptom onset and age at diagnosis, which were captured in months for children aged ≤ 1 year, in half years for those participants aged ≤ 4 years, and in full years for children > 5 years. Time from symptom onset to diagnosis was then calculated using these ages. The two time to diagnosis measures had a high linear (Pearson) correlation, 80% (95%CI 75%, 85%). Given that the time from actively seeking treatment was more granular, this was chosen as our measure of time to diagnosis. Based on previous systematic review [14], and clinical feedback from pediatric rheumatologists on the window of opportunity to prevent long-term joint damage and disability, it was decided to compare outcomes for children diagnosed < 6 months versus 6 + months from actively seeking treatment.

### Outcome measures

Health-related quality of life (HRQoL) was assessed using the Child Health Utility Instrument (CHU9D) [19]. CHU9D uses a 5-point scale across nine specific domains. Australian preference weights were used to transform CHU9D scores into utility values ranging between 1.00 (perfect health) to zero (being dead) [20]. Utility values allowed for the estimation of Quality-Adjusted Life Years (QALYs) for participants, enabling cost-utility and cost-benefit analyses to be conducted.

Annual government healthcare costs were estimated based on participant responses to the IMPACT survey on healthcare use, including medical investigations, health professional visits, hospitalizations and medications. Costs were estimated by applying Australian government subsidy item costs (medications) [21], schedule fees (medical services) [22], and National Efficient Prices (hospital stays) [23]. The net benefit in dollars was estimated using the present value of incremental healthcare costs plus the willingness to pay for one QALY multiplied by the present value of incremental QALYs.

### Analysis

#### Statistical analysis

Descriptive statistics were calculated and comparisons made between sociodemographic and clinical characteristics of survey participants by time to diagnosis (< 6 months versus 6 + months) using chi-squared tests for categorical data and t-tests for continuous variables. Linear regression was conducted to estimate the relationship between time to diagnosis and the main outcome measures of HRQoL and annual government healthcare costs. Given the cross-sectional nature of the survey, additional analysis was conducted using multivariable generalized additive models to take into account non-linearity of age, and potential confounding by age, gender and time from diagnosis to survey completion and potential interaction by age-gender or time from diagnosis to survey completion and time to diagnosis to ensure inferences were robust.

#### Economic analysis

A cost-utility analysis was conducted over a lifetime horizon from a health funder perspective in 2023 Australian dollars, comparing time from actively seeking treatment to diagnosis (< 6 months, 6 + months). A lifetime horizon was chosen for this analysis, given that those with delayed diagnosis were more likely to suffer lifelong disability, less likely to gain treatment-free remission, and more likely to require care into adulthood [7]. Each participant’s Quality Adjusted Life Years (QALYs) were estimated using their life expectancy [21] and multiplied by their CHU9D utility value. Costs and QALYs were present valued over a lifetime horizon using a 5% per annum discount rate [24]. The net benefit in dollars per child of an earlier diagnosis was calculated using each participant’s present valued QALYs and health system costs, and the generally accepted willingness to pay of $50,000 for one QALY in Australia [25]. One-way sensitivity analyses were also conducted for robustness: (1) Discount rates 3% and 7%, (2) Willingness to pay for one QALY ($30,000, $70,000), and (3) Removing participants completing the survey within one year of diagnosis to remove any short-term treatment effects on HRQoL and costs within the first year of treatment. A pre-defined economic analysis plan was followed (Additional file 1), and the analysis is reported in accordance with the Consolidated Health Economic Evaluation Reporting Standards (CHEERS) checklist [26] (Additional file 2). All statistical and economic analyses were performed using R (ver. 4.4.1)(R Core Team 2023) [27] packages Gmisc for plot and table output [28], forestplot [29] and knitr (Xie 2023) [30] for reproducible research and results with P-values < 0.05 were considered statistically significant.

The study was approved by The University of Sydney Human Research Ethics Committee (HREC: 2022/902).

## Results

Of the 184 IMPACT survey participants with JIA, 163 (89%) were included based on those who had completed the CHU9D instrument. Participants missing CHU9D were almost equally distributed between the time to diagnosis groups (< 6 months: *n* = 14/116 (12%); 6 + months: *n* = 7/68 (10%)) with no systematic difference in characteristics to those with completed CHU9D. Survey participants ranged from having been just diagnosed to 21 years after diagnosis, but were concentrated in the first five years post-diagnosis, median (interquartile range) 4.5 (1.9–8.4) years (Table 1). Most importantly, the time from diagnosis to completing the survey did not differ significantly between diagnosis groups (*p* = 0.14) (Table 1). Compared to those diagnosed 6 + months after actively seeking treatment, those diagnosed < 6 months were younger at symptom onset, mean(SD) 4.4(3.8) versus 5.7(3.6) years; *p* = 0.03, at diagnosis, and at survey completion, mean(SD) 11.0 (5.2) versus 12.8 (4.8), *p* = 0.03 (Table 1). There was no evidence of a relationship between time to diagnosis and gender, ethnicity, location (state/territory), parental education, socioeconomic status, rurality or any other clinical condition (all *p* > 0.05), except uveitis (*p* = 0.001), which is known to be positively associated with an earlier diagnosis [14] ] (Table 1).

**Table 1:** Demographic and clinical characteristics of survey participants by time to diagnosis

Demographic and clinical characteristics	Time to diagnosis in months		Total *N* = 163	*P* value^**^
	< 6 *N* = 102	6 + *N* = 61		
**Age at symptom onset in years**				**0.028**
Mean (SD)	4.4 (± 3.8)	5.7 (± 3.6)	4.9 (± 3.8)	
Median (IQR)	3.0 (1.5–6.0)	5.0 (2.0–9.0)	4.0 (2.0–7.0)	
**Age at diagnosis in years**				**< 0.001**
Mean (SD)	4.7 (± 3.9)	7.6 (± 3.8)	5.8 (± 4.1)	
Median (IQR)	3.0 (2.0–6.0)	7.0 (4.0–11.0)	5.0 (2.0–9.0)	
**Age at survey in years**				**0.032**
Mean (SD)	11.0 (± 5.2)	12.8 (± 4.8)	11.7 (± 5.1)	
Median (IQR)	10.3 (7.0–14.9)	12.9 (9.5–16.0)	11.8 (7.6–15.5)	
**Time from diagnosis to completing survey in years**				**0.14**
Mean (SD)	6.3 (± 4.9)	5.1 (± 4.8)	5.8 (± 4.9)	
Median (IQR)	5.6 (1.8–8.6)	3.4 (1.9–5.9)	4.5 (1.9–8.4)	
**Gender**				**0.78**
Male	30 (29.4%)	20 (32.8%)	50 (30.7%)	
Female	72 (70.6%)	41 (67.2%)	113 (69.3%)	
**Ethnicity**				**0.37**
White/European	82 (80.4%)	53 (86.9%)	135 (82.8%)	
Aboriginal Australian/Torres Strait Islander	4 (3.9%)	1 (1.6%)	5 (3.1%)	
Asian	5 (4.9%)	5 (8.2%)	10 (6.1%)	
Middle Eastern	7 (6.9%)	1 (1.6%)	8 (4.9%)	
Other	4 (3.9%)	1 (1.6%)	5 (3.1%)	
**Australian State or Territory**				**0.36**
Australian Capital Territory	4 (3.9%)	3 (4.9%)	7 (4.3%)	
New South Wales	62 (60.8%)	29 (47.5%)	91 (55.8%)	
Queensland	10 (9.8%)	12 (19.7%)	22 (13.5%)	
South Australia	11 (10.8%)	5 (8.2%)	16 (9.8%)	
Tasmania	1 (1.0%)	2 (3.3%)	3 (1.8%)	
South Australia	7 (6.9%)	7 (11.5%)	14 (8.6%)	
Western Australia	7 (6.9%)	3 (4.9%)	10 (6.1%)	
**Parental education (highest level)**				**0.55**
Did not complete high school	4 (3.9%)	3 (4.9%)	7 (4.3%)	
HSC or equivalent (Year 12)	14 (13.7%)	12 (19.7%)	26 (16.0%)	
College/TAFE/Diploma	28 (27.5%)	14 (23.0%)	42 (25.8%)	
University	25 (24.5%)	19 (31.1%)	44 (27.0%)	
Postgraduate	31 (30.4%)	13 (21.3%)	44 (27.0%)	
^*^ **SEIFA Quintiles - IRSD**				**0.35**
1 - Most disadvantaged	14 (13.7%)	10 (16.4%)	24 (14.7%)	
2	19 (18.6%)	9 (14.8%)	28 (17.2%)	
3	22 (21.6%)	15 (24.6%)	37 (22.7%)	
4	23 (22.5%)	7 (11.5%)	30 (18.4%)	
5 - Least disadvantaged	24 (23.5%)	20 (32.8%)	44 (27.0%)	
^**†**^ **Remoteness**				**0.27**
Major city	81 (79.4%)	42 (68.9%)	123 (75.5%)	
Inner regional	16 (15.7%)	13 (21.3%)	29 (17.8%)	
Outer regional	5 (4.9%)	6 (9.8%)	11 (6.7%)	
**Private health insurance**				**0.99**
Yes	62 (60.8%)	37 (60.7%)	99 (60.7%)	
No	40 (39.2%)	24 (39.3%)	64 (39.3%)	
**Other reported clinical conditions**				
Bone fractures	9 (8.8%)	12 (19.7%)	21 (12.9%)	**0.079**
Dental problems	18 (17.6%)	16 (26.2%)	34 (20.9%)	**0.27**
Diabetes	2 (2.0%)	1 (1.6%)	3 (1.8%)	**0.99**
^ ﻿¶^Heart problems	5 (4.9%)	5 (8.2%)	10 (6.1%)	**0.61**
Kidney	3 (2.9%)	1 (1.6%)	4 (2.5%)	**0.99**
Psoriasis	7 (6.9%)	11 (18.0%)	18 (11.0%)	**0.052**
Uveitis	34 (33.3%)	6 (9.8%)	40 (24.5%)	**0.001**

^*^Socio-economic indices Australia, 2021, quintiles derived from national deciles by participant’s postal area, IRSD - Index of Relative Socio-economic Disadvantage

^†^Remoteness measured by Accessibility/ Remoteness Index of Australia Plus (ARIA+) based on participant’s postal area

^¶^Pericarditis, myocarditis, endocarditis, ventricular dysfunction

^**^P values are chi-squared tests for proportions and t-tests for continuous variables

The average CHU9D score was lower for children diagnosed at 6 + months, 0.44 (SD 0.29), compared to those diagnosed < 6 months, 0.59 (SD 0.29); *p* = 0.003 (Table 2). Within the nine dimensions that comprise the CHU9D, children in the < 6 months diagnosis group reported higher scores in the dimensions of pain, tiredness, sleep, daily routine, and activities than those in the delayed diagnosis group (*p* < 0.05) (Table 2).

**Table 2:** Child Health Utility 9D Instrument (CHU9D) Dimension Scores by time to diagnosis groups

Child Health Utility Instrument (CHU9D) Dimension Scores Utility Value (Range: 1 = Perfect health, 0 = Dead)	Time to diagnosis in months		t-Test *P* value
	< 6 *N* = 102	6 + *N* = 61	
	Mean (SD)		
Overall*	0.59 (± 0.29)	0.44 (± 0.29)	0.003
**HRQoL dimensions**			
Worry (Range 0.11 to 0.22)	0.17 (± 0.05)	0.15 (± 0.05)	0.11
Sadness (Range 0.02 to 0.15)	0.10 (± 0.05)	0.09 (± 0.05)	0.07
Pain* (Range − 0.02 to 0.11)	0.05 (± 0.04)	0.02 (± 0.04)	0.001
Tiredness* (Range 0.03 to 0.11)	0.07 (± 0.02)	0.06 (± 0.02)	0.001
Annoyance (Range − 0.04 to 0.08)	0.04 (± 0.04)	0.03 (± 0.04)	0.09
School (Range − 0.02 to 0.09)	0.05 (± 0.04)	0.04 (± 0.04)	0.07
Sleep* (Range − 0.05 to 0.06)	0.03 (± 0.03)	0.02 (± 0.03)	0.03
Daily routine* (Range − 0.05 to 0.07)	0.02 (± 0.05)	-0.00 (± 0.05)	0.008
Activities* (Range 0.02 to 0.13)	0.11 (± 0.02)	0.10 (± 0.03)	0.007

*Statistically significant at *p* = 0.05

Compared with 6 + months, a time to diagnosis < 6 months was associated with a clinically and statistically significant increase in HRQoL utility value of 0.14 (95%CI 0.05, 0.23) per child (Table 3). Results were also comparable after adjusting for potential confounding and interaction by age, sex and time from diagnosis to survey completion, 0.14 (95%CI 0.04, 0.23) (Table 3).

**Table 3:** Model estimates for Health-related Quality of Life (HRQoL) measured by Child Health Utility 9D Instrument (CHU9D) Utility Value by time to diagnosis group

Child Health Utility Instrument (CHU9D) Utility Value (Range: 1 = Perfect health, 0 = Dead)	Time to diagnosis in months		Difference	Wald Test *P* value
	< 6 *N* = 102	6 + *N* = 61		
	Mean (95% CI)			
Unadjusted	0.59 (0.53, 0.64)	0.44 (0.39, 0.52)	⇑0.14 (0.05, 0.23)	0.003
Adjusted^*^	0.58 (0.50, 0.67)	0.45 (0.35, 0.55)	⇑0.14 (0.04, 0.23)	0.004

*Adjusted by age, gender, and age-gender interaction. There was no evidence that time from diagnosis to the survey (Wald Test P value = 0.193), or an interaction between diagnosis group and time from diagnosis to the survey (Likelihood Ratio Test P value = 0.112), improved model fit

The average annual government healthcare costs for children diagnosed at <6months was $18,765 (SD $6,416) and lower than the average annual cost of $29,494 (SD $37,034) for children diagnosed 6 + months (*p* = 0.012) (Table 4). Large cost savings were observed for the < 6 months group in overall hospitalizations, and those specifically for surgeries, pain, inflammation, and investigative procedures (Table 4). Smaller, but significant cost savings were also found in fewer visits to primary care physicians and allied health professionals (Table 4).

**Table 4:** Annual government healthcare costs (medical investigations, health professional visits, hospitalizations, and medications) related to JIA in Australian dollars ($) by time to diagnosis group

*Annual* government healthcare costs in Australian dollars ($)	Time to diagnosis in months		t-Test *P* value
	< 6 *N* = 102	6 + *N* = 61	
	Mean (SD)		
**Total costs**	**18**,**765 (± 16**,**416)**	**29**,**494 (± 37**,**034)**	**0.012**
***Cost components***			
**Medical investigations** ^*^			
X-rays, blood tests, MRI, Ultrasound, CT scans, nuclear medicine, eye examinations	1,694 (± 621)	1,753 (± 824)	0.60
***Subtotal***	***1***,***694 (± 621)***	***1***,***753 (± 824)***	***0.60***
**Health professional visits** ^*^			
Primary care physician^*†^	459 (± 230)	575 (± 295)	0.005
Clinical specialists^*^	984 (± 1132)	1,080 (± 863)	0.57
Rheumatology Nurse	464 (± 791)	421 (± 1007)	0.76
Allied health professionals	1,153 (± 1649)	1,831 (± 2203)	0.03
***Subtotal***	***2***,***596 (± 2***,***754)***	***3***,***487 (± 2***,***976)***	***0.05***
**Hospitalizations**			
Bone fracture	-	480 (± 3751)	-
Infection/virus	811 (± 4,262)	2,612 (± 10,164)	0.12
Injections/infusions	4,750 (± 8,358)	5,657 (± 9,543)	0.53
Mental health	398 (± 4,015)	-	-
Pain/inflammation/investigative	1,510 (± 4,524)	7,299 (± 26,726)	0.03
Surgery	28 (± 288)	281 (± 1,071)	0.03
***Subtotal***	***7***,***498 (± 12***,***152)***	***16***,***329 (± 31***,***275)***	***0.01***
**Medications** ^**^			
^ §^csDMARDS	397 (± 242)	430 (± 315)	0.46
^ ¶^bDMARDS	5,489 (± 6033)	6,080 (± 6,980)	0.57
^ ∥^tsDMARDS	943 (± 4,176)	1,262 (± 4,804)	0.66
^ #^Corticosteriod ORAL	148 (± 189)	153 (± 191)	0.89
***Subtotal***	***6***,***978 (± 7***,***446)***	***7***,***925 (± 9***,***549)***	***0.48***

^*^Applies the Medicare Safety Net as indicated by the participant

†Assumes participants visiting allied health completed a chronic disease care plan with primary physician

‡Assumes chronic disease plan rebates are claimed

§Assumes dosages: Methotrexate Oral and Subcutaneously 15 mg per week (60% participants oral, 40% injections), Hydroxychloroquine 200 mg daily, Suflasalazine 1000 mg daily, Leflunomide 20 mg daily

¶Assumes dosages: Adalimumab 40 mg/0.8mL injection every 2 weeks, etanercept 50 mg/mL injection weekly, Infliximab 120 mg/mL injection monthly, Tocilizumab 162 mg/0.9 mL injection weekly, Abatacept 125 mg/mL injection weekly, Secukinumab 150 mg/mL injection weekly

∥Assumes dosages: Tofacitinib 5 mg twice daily, Upadacitinib 15 mg daily, Baricitinib 4 mg daily

#Assumes dosages: Prednisone 25 mg daily

**Excludes all other medications than DMARDS or ORAL corticosteriods. Note injections and infusions of corticosteriods are assumed to be included in hospitalisation costs

Overall, this represented significant annual savings in healthcare costs for the < 6 months versus 6 + months group of $10,729 (95%CI $2,393, $19,065); *p* = 0.01 (Table 5).

**Table 5:** Model estimates for annual government healthcare costs (medical tests, health professional, hospital, and medications) related to JIA in Australian dollars ($) by time to diagnosis group

*Annual* government healthcare costs in Australian dollars ($)	Time to diagnosis in months		Difference	Wald Test *P* value
	< 6 *N* = 102	6 + *N* = 61		
	Mean (95% CI)			
Unadjusted^*^	18,765 (13,665, 23,864)	29,494 (22,899, 36,088)	⇓-10,729 (-2,393, -19,065)	0.012

^*^Adjusted estimates are not shown as there was no evidence of a difference in annual government healthcare costs by age (Wald Test, P value = 0.536), gender (Wald Test P value = 0.899), or age-gender interaction (Likelihood Ratio Test P value = 0.103). In addition, there was no evidence that adding time to diagnosis (Wald Test P value = 0.651), or an interaction term between diagnosis group and time from diagnosis to the survey (Likelihood Ratio Test P value = 0.289), improved model fit

**Fig. 1:**
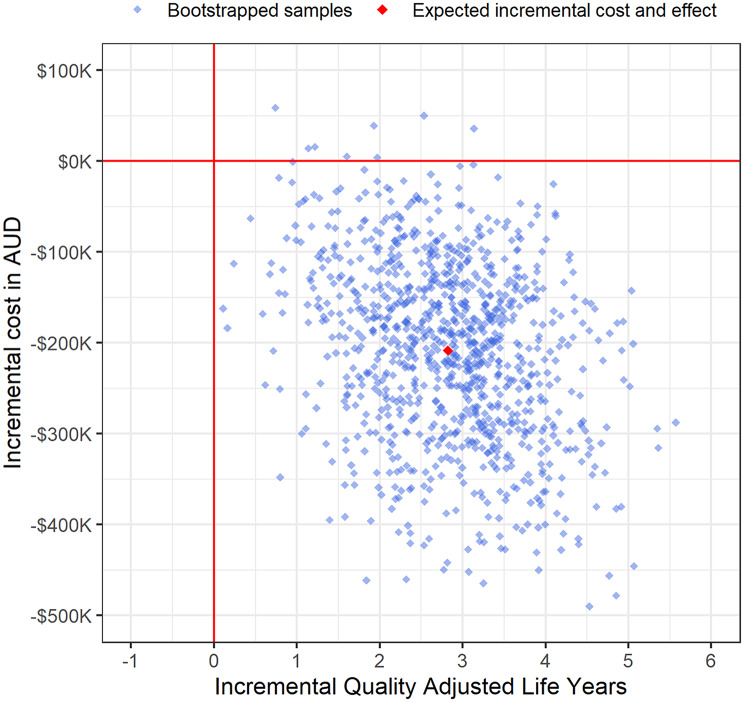
Cost effectiveness plane: Incremental healthcare costs in Australian dollars (AUD) per quality adjusted life years saved from JIA diagnosis within six months of actively seeking treatment

Over a lifetime horizon, the present value of healthcare savings from diagnosis within six months was $208,458 (95%CI $45,388, $371,528); *p* = 0.01, and QALYs increased by 2.82 (95%CI 1.03, 4.61);*p* = 0.002 per child (Table 6; Fig. 1). Using the generally accepted willingness-to-pay of $50K per QALY [25], the net benefit to the Australian government (health funder) of diagnosis within the 6-month window was $349,520 (95%CI $139,630, $559,411);*p* = 0.001 per child with JIA (USD$231,470 (95%CI $92,470, $370,471) [31])(Table 6).

**Table 6:** Lifetime change in government healthcare costs, Quality Adjusted Life Years and Net Benefit in Australian and US Dollars ($) from diagnosis within six months of actively seeking treatment

Cost-benefit	Time to diagnosis in months < 6 versus 6+ Mean difference (95%CI)
Healthcare savings in AUD	⇑$208,458 (95%CI $45,388, $371,528); *p* = 0.01
Quality Adjusted Life Years (QALYs)	⇑2.82 (95%CI 1.03, 4.61);*p* = 0.002
Net Benefit @ $50K per QALY in AUD	⇑$349,520 (95%CI $139,630, $559,411);*p* = 0.001
Net Benefit @ $50K per QALY in USD	⇑$231,470 (95%CI $92,470, $370,471);*p* = 0.001

AUD = Australian Dollars, USD = US Dollars

Annual healthcare use, including medical investigations, health professional visits, hospitalizations and medications, was determined using data from participants completing The IMPACT Survey [18]. Costs were estimated by applying Australian government subsidy item costs (medications) [21], schedule fees (medical services) [22], and National Efficient Prices (hospital stays) [23]. Each participant’s Quality Adjusted Life Years (QALYs) were estimated using their life expectancy [21] multiplied by their CHU9D utility value. Costs and QALYs were present valued over a lifetime horizon using a 5% per annum discount rate [24]. 2023 AUD/USD Exchange rate 1.51 [31]

**Fig. 2:**
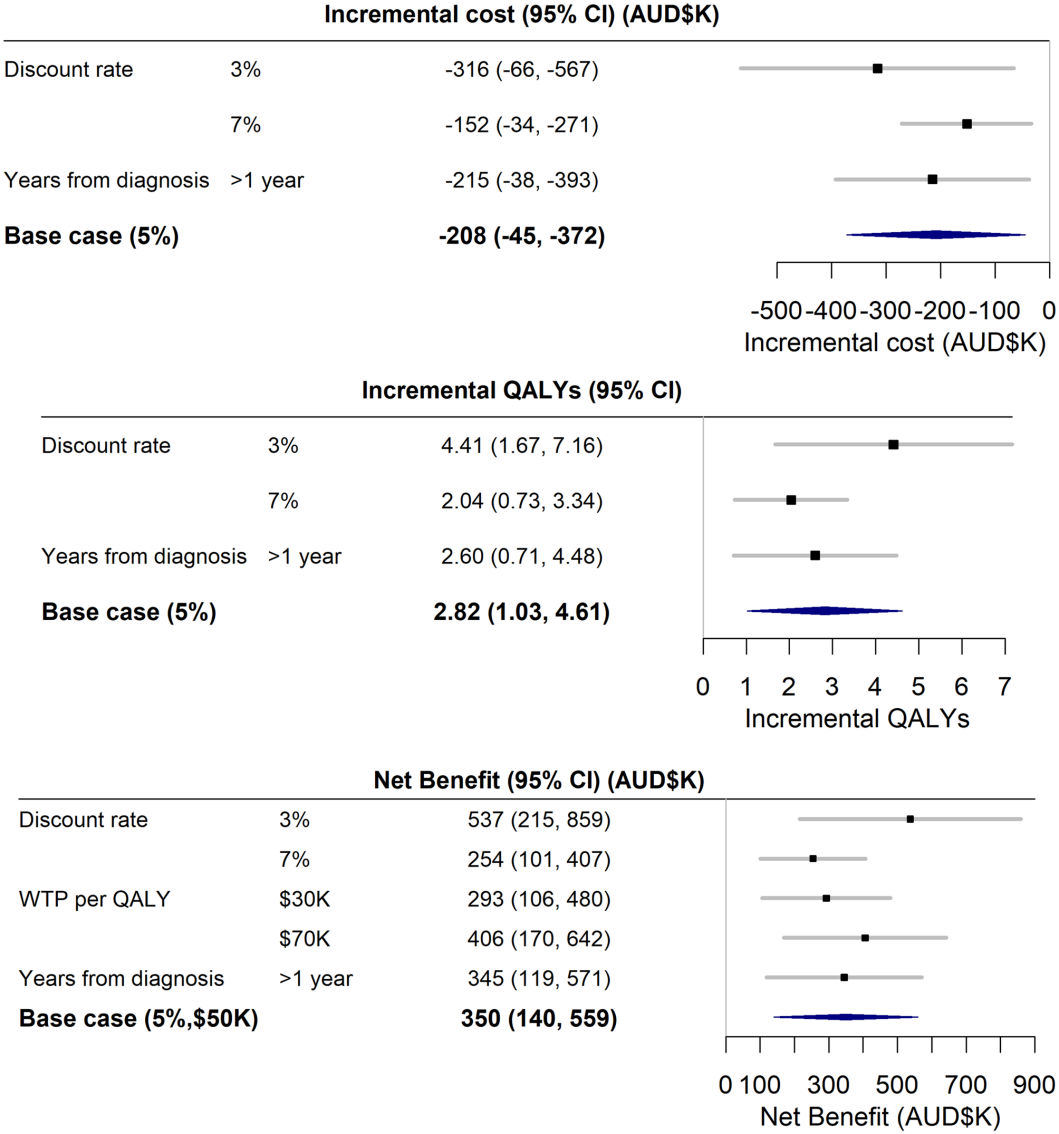
One-way sensitivity analysis forestplot

One-way sensitivity analysis showed that the net benefit was most sensitive to the assumed time value of money, with the net benefit reduced to $254K at a 7% discount rate and increased to $537K at a 3% discount rate (Fig. 2). Removing potential short-term treatment effects by reperforming the analysis excluding the 17 participants who completed the survey within one year of diagnosis, resulted in a negligible reduction in the net benefit of $5K (1.4%)(Fig. 2).

The net benefit in dollars per child with JIA can be used to set the cost threshold per child for an intervention to be cost-effective, value the benefits of alternate interventions, or estimate the total budget for interventions using the benefits forgone of not improving time to diagnosis. For example, in Australia, based on the estimated 5.1 million [32] resident children under 16 years as at 30 June 2023, and using a very conservative annual incidence based on children with a first admission to public hospitals of 7 per 100,000 [33] (358 children), and 37% of children receiving a diagnosis 6 + months from actively seeking treatment, the total benefits forgone of delayed diagnosis to healthcare funders for those 134 children would be $47 (95%CI $19, $75) million.

## Discussion

The IMPACT Survey [18] confirmed little has changed in Australia in terms of time to diagnosis from 2014 to 2023 (6 + month waiting time: 41% of children in 2014 [1] versus 37% in 2023 [18]). Compared to children diagnosed within 6 months of actively seeking treatment, children waiting 6 + months for diagnosis reported lower health-related quality of life, with deficits in the dimensions of pain, tiredness, sleep, daily routine and activities. The difference did not diminish over the years from diagnosis (participant data reached 20 years post diagnosis), supporting the hypothesis of a window of opportunity for early aggressive treatment with disease-modifying drugs to limit long-term joint damage and disability [6–9]. Children in the delayed diagnosis group also reported a higher usage of health services, resulting in an average increase in costs for the healthcare funder of more than $10,000 per child. The majority of this cost increase was from hospitalizations for pain, inflammation, and investigative procedures. This and the fact that the higher health service use did not diminish in the years following diagnosis (participant data reached 20 years post diagnosis) again supports the hypothesis of a window of opportunity for early aggressive treatment.

As the HRQoL and healthcare cost differences from time to diagnosis are likely to persist over a lifetime, the present value of Quality Adjusted Life Years (QALY) saved, 2.8 (95%CI 1.0, 4.6), and healthcare cost savings, $208,458 (95%CI $45,388, $371,528), from diagnosis within six months of actively seeking treatment are sizeable. Assuming a willingness to pay of $50K per QALY saved, the net benefit to the healthcare funder is estimated at $350K (95%CI $140K, $559K) per child with JIA. Therefore, a $350K intervention to improve just one child’s time to diagnosis within six months would likely be cost-effective for the healthcare funder. Since there is a significant annual incidence of JIA, and 37 out of each 100 children and adolescents with incident JIA are impacted by a delayed diagnosis in Australia, an intervention to improve times to diagnosis has the potential to have a sizeable budget impact based on the number of children assisted.

To our knowledge, this is the first study to quantify the health economic impact of delayed diagnosis of JIA. Studies of autism have shown similar health benefits and healthcare cost savings with early diagnosis. The early identification of autism allows for timely interventions that significantly improve developmental outcomes, which can reduce the cost of special education services in childhood [34], and reduce dependency in adulthood [35].

There are a number of opportunities to reduce the time to diagnosis. In 2014, Arthritis Australia concluded that inadequate public and health practitioner awareness of the condition and limited access to specialists for diagnosis and care appear to be key factors leading to these delays [1], and these factors persist today, with the longest delay from the first healthcare professional visit to PR referral [16]. Knowledge of JIA amongst primary health physicians, pediatricians and other specialists is an issue internationally [36]. In Europe and North America, several organizations are devoting considerable efforts to increasing knowledge of JIA among primary healthcare providers [36], while in Australia, a nationwide communication strategy to promote early diagnosis of JIA has been rolled out by the consumer group Juvenile Arthritis Foundation Australia, through professional organisations and Primary Health Networks, highlighting early symptoms of JIA [37].

The waiting time from referral to appointment with a PR may also impact time to diagnosis. Paediatric rheumatology services in Australia and New Zealand fall well behind accepted international benchmarks, with a significant shortfall in the number of PRs [38]. There is excessive reliance on services provided in private practice, and the shortage of publicly funded PRs is a major barrier to providing timely and accessible care for children, particularly those living in regional and rural areas [39]. It is interesting to note that the hiring of an additional public PR would cost the healthcare funder less than $350K and would likely assist more than one additional child to diagnosis within six months, making it a cost-effective intervention.

The strengths of this study include the design of survey questions with input from patients, families, and clinicians, the use of child-specific, validated HRQoL instruments with population-appropriate utility value sets, the collection of detailed healthcare usage data, and the use of robust and consistent standard government unit prices for cost estimates.

Study limitations include the usual cross-sectional survey biases, such as selection bias and confounding. However, the age, gender, and socio-economic groups represented in the study were representative of the JIA population, and thorough testing and adjustment for potential confounders were performed. Recall bias for time to diagnosis and healthcare usage is another limitation. However, our findings regarding the months of delay in diagnosis and overall healthcare costs align with international literature. The assumption of a constant difference in HRQoL and health system costs between diagnosis groups over a lifetime horizon is a limitation. However, it is a conservative assumption, given longitudinal studies suggest that HRQoL differences and health system costs are likely persist or worsen over time [10–12, 40, 41]. In addition, removing potential short-term treatment effects by reperforming the analysis excluding the 17 participants who completed the survey within one year of diagnosis, resulted in a negligible reduction in the net benefit of $5K (1.4%).

### Policy implications

In the era of disease-modifying medications that, with early aggressive treatment, can prevent long-term joint damage and disability in children with JIA, international estimates that 50% of children with JIA are waiting six months or more from symptom onset for diagnosis and treatment [14] represents a significant cost in terms of health economic benefits forgone. This article provides health funders and policymakers with relevant estimates of the delay in diagnosis in terms of HRQoL, QALYs, and health system costs. An intervention to improve the time to diagnosis of JIA within six months is expected to have a present value of $350K (US$231K) per child with JIA in terms of QALYs saved and health system costs at a generally accepted willingness to pay for one QALY of $50K. In the context of a significant annual incidence of JIA [3, 4], and 50 out of 100 children with incident JIA waiting six months or more for diagnosis [14], an intervention to improve time to diagnosis has the potential to have a sizeable budget impact based on the number of children assisted. Not only are the health economic benefits sizeable, but the improvement in HRQoL for people living with JIA is beneficial.

## Conclusion

Interventions to improve the time to diagnosis of JIA within six months of seeking help are likely cost-effective and can significantly improve HRQoL for people living with JIA.

## Supplementary Information

Below is the link to the electronic supplementary material.

Supplementary Material 1: Additional file 1, DOC (Microsoft Word), Economic Analysis Plan, Details of the pre-defined Economic Analysis Plan

Supplementary Material 2: Additional file 2, DOC (Microsoft Word), CHEERS Checklist, Completed CHEERS Checklist for the study

## Data Availability

Data are available upon reasonable requests and discussion with the authors.

## Abbreviations

AUD: Australian dollars
95% CI: 95% Confidence Interval
CHU9D: Child Health Utility 9D instrument
HRQoL: Health-Related Quality of Life
IQR: Interquartile Range
JIA: Juvenile Idiopathic Arthritis
PR: Pediatric Rheumatologist
QALYs: Quality-Adjusted Life Years
USD: United States Dollars
