# Supplementary material for: The impact of time to diagnosis on health service use, cost, and quality of life for patients with juvenile idiopathic arthritis: a cost-utility analysis
Source: Pediatr Rheumatol Online J. 2026 Feb 24;24:12. doi: 10.1186/s12969-026-01195-7 (PMC12937577; doi:10.1186/s12969-026-01195-7)
Supplement: Supplementary file 1 — Supplementary Material 1: Additional file 1, DOC (Microsoft Word), Economic Analysis Plan, Details of the pre-defined Economic Analysis Plan [file 12969_2026_1195_MOESM1_ESM.docx]

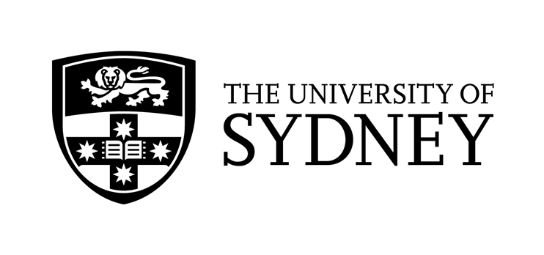


Medical

**The Cost of Living with Juvenile Idiopathic Arthritis**

To quantify the relationship between time to diagnosis and the impact on child HRQoL and estimate the net benefit in dollars to healthcare funders of an earlier time to diagnosis

**ECONOMIC EVALUATION PLAN**

Prepared by: *Amy von Huben* ***Study Health Economist***

**Date: 2 September 2025**


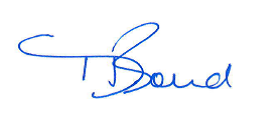


Signed by:

***Principal Investigator(s)***: Dr Diana Bond *Date: 05/09/2025*

Contents

[1 Economic evaluation design 4](#_Toc207706057)

[1.1 Background 4](#_Toc207706058)

[1.2 Intervention 4](#_Toc207706059)

[1.3 Control 4](#_Toc207706060)

[1.4 . Main objective 4](#_Toc207706061)

[1.5 Sample size 4](#_Toc207706062)

[1.6 Schedule of Data Collection 4](#_Toc207706063)

[2 PATIENT POPULATION 6](#_Toc207706064)

[2.1 Inclusion criterion 6](#_Toc207706065)

[3 ANALYSIS 6](#_Toc207706066)

[3.1 Analysis methodology 6](#_Toc207706067)

[3.2 Data flow chart 7](#_Toc207706068)

[3.3 Demographics 7](#_Toc207706069)

[3.4 Costs 9](#_Toc207706070)

[3.5 Quality of Life 13](#_Toc207706071)

[3.6 Effectiveness measure 13](#_Toc207706072)

[3.7 Cost measure 13](#_Toc207706073)

[3.8 Main outcomes 14](#_Toc207706074)

[3.9 One way sensitivity analyses 14](#_Toc207706075)

List of Tables

[Table 1: Schedule of data collection 5](#_Toc208407267)

[Table 2 Patient and clinical characteristics 8](#_Toc208407268)

[Table 3: Medications in the last year 10](#_Toc208407269)

[Table 4: Medical investigations in last past year 11](#_Toc208407270)

[Table 5: Hospitalisation admissions in the last year by state 11](#_Toc208407271)

[Table 6: Medical professional visits in last year 11](#_Toc208407272)

[Table 7: Annual government healthcare costs (medical investigations, health professional, hospital, and medications) related to JIA for the participants over the last 12 months 13](#_Toc208407273)

[Table 8 Child Quality of Life as measured by Child Health Utility (CHU9D)- Dimension scores 14](#_Toc208407274)

[Table 9 Child Quality of Life as measured by Child Health Utility (CHU9D) utility values 14](#_Toc208407275)

[Table 10 Effectiveness measures 14](#_Toc208407276)

[Table 11 Cost measures 15](#_Toc208407277)

[Table 12: Net benefit of diagnosis within 6 months 15](#_Toc208407278)

List of Figures

[Figure 1 Cost-effectiveness plane for the main objective 15](#_Toc207706092)

Abbreviations

| SD | Standard Deviation |
| --- | --- |
| IQR | Interquartile range |
| CI | Confidence Interval |
| n | Number in that cell |
| HRQoL | Health -related Quality of Life |
| CHU9D | Child Health Utility 9 Dimensions |
| ICER | Incremental Cost-Effectiveness Ratio |
| QALY | Quality Adjusted Life Year |
| WTP | Willingness to Pay |
| JIA | Juvenile Idiopathic Arthritis |
| AUD | Australian dollar |

# Economic evaluation design

This economic evaluation is a cost-utility analysis (CUA) and cost-benefit analysis (CBA) over a life-time horizon in 2023 Australian dollars from the perspective of the health funder, the Australian Commonwealth Government.

## Background

An online prospective survey administered via REDCap, the IMPACT study, will examine lived experiences of children and families in Australia. The survey was publicly available from February 16, 2023 (with a reminder released on March 18, 2023) until 30 June, 2023. The data from this survey on financial costs and health-related quality of life will be used for the economic evaluation.

## Intervention

Time from actively seeking treatment to diagnosis (“time to diagnosis”) less than 6 months

## Control

Time from actively seeking treatment to diagnosis (“time to diagnosis”) 6 months or more.

## . Main objective

The aim of this study is two-fold:

1. To quantify the relationship between time to diagnosis and the impact on child HRQoL

2. Estimate the net benefit in dollars to healthcare funders of an earlier time to diagnosis.

The net benefit in dollars will be used to quantify the budget available for cost-effective interventions to improve times to diagnosis (i.e., threshold analysis). Net benefit is defined as willingness to pay for one QALY multiplied by QALYs saved plus heath system savings.

## Sample size

Sample will be determined by the number of completed surveys received by the IMPACT study for participants with JIA.

## Schedule of Data Collection

Data will be collected by a self-administered online prospective survey administered via REDCap at one point in time (see Table 1).

Table 1: Schedule of data collection

| Data items | Date (period) |
| --- | --- |
| 1. Demographic information including age, sex, ATSI status, postcode, education and health insurance. | X |
| 1. Diagnostic information and related co-morbidities | X |
| 1. Quality of life measuring physical, social and emotional wellbeing will be assessed using the following validated paediatric measures that have been previously utilised in similar populations and age groups:    1. visual analog scale (VAS)^28^    2. age-specific PedQL & Arthritis module^30^    3. CHU9 | X |
| 1. Assessment of practical and financial impacts of JA including out-of-pocket healthcare costs, travel, parking, childcare, time-off work, medications, private specialist/allied health visits, investigations, practical aids and employment. These questions are based on adaptations of previous surveys for Diabetes^31^ and Chronic Kidney Disease^32^ and a validated Health and Labour Questionnaire.^33^ | X |

# PATIENT POPULATION

## Inclusion criterion

The study population will include:

- Parents of individuals up to (and including) 25 years of age
- Adults from 18-25 years of age

who have been diagnosed with Juvenile Idiopathic Arthritis.

Participants must be able to read and write in English and have access to internet for survey completion. Must live in Australia.

# ANALYSIS

## Analysis methodology

Descriptive statistics of demographic characteristics, age at diagnosis, other health conditions, medications and treatment, visits to health care professionals will be determined by time to diagnosis groups. Descriptive results will be presented as percentages and counts for discrete variables, and as mean and standard deviation or median and interquartile range for quantitative variables. Proportions tested with chi-square test or log binomial regression if adjustment is required for characteristics differences. Continuous outcomes will be compared with t-tests or linear regression for adjustments to baseline unbalance. A Mann-Whitney test will be considered if the distributional assumptions for t-test and linear regression are not met. For ordinal outcomes, we will use Mann-Whitney tests or ordinal logistic regression if adjustment required for baseline differences.

Health-related quality of life (HRQoL) will be measured using the Child Health Utility instrument (CHU9D)^1.^ CHU9D uses a 5-point scale across nine specific domains. Australian preference weights will be used to transform CHU9D scores into utility values ranging between 1.00 (perfect health) to zero (being dead)^2^. Utility values allow for the estimation of Quality-Adjusted Life Years (QALYs) and enable the cost-utility analysis to be conducted. Each participant's Quality Adjusted Life Years (QALYs) will be estimated using their life expectancy^3^ discounted by 5% per annum^4^ and multiplied by their CHU9D utility value.

Annual healthcare use, including medications, health professional/hospital visits, and medical investigations will determined using data from participants completing the survey. The corresponding costs of medications will be calculated based on the number of standard units and dosage of medication specified and number of standard packs required over an annual period. Costs will be estimated by applying Australian government subsidy item costs (medications)^3,^ schedule fees (medical services)^5^, and National Efficient Prices (hospital stays)^6^. Out-of-pocket expenses will not be included in this analysis as the evaluation is from a health funder perspective.

All the tests will be two-tailed and a significance level of 0.05 will be used. No correction for multiple comparisons will be made for the analyses. All analyses will be conducted using R. Missing data will be multiple imputed, where appropriate.

Patient-level data on costs and QALYs will be aggregated and presented in total and average for both time to diagnosis groups. Incremental costs and incremental benefits of the intervention (timely diagnosis) group will be calculated from the difference in means of the two time to diagnosis groups and presented with a 95% Confidence Interval (CI). Incremental cost-effectiveness ratios (ICERs) will be calculated. Using a bootstrap procedure to estimate uncertainty, a 95% CI for the incremental cost-effectiveness ratio (ICER) will be calculated, and estimates will be plotted on a cost-effectiveness plane. The result will be transformed into a net benefit in dollars to healthcare funders using $50,000 as the willingness to pay for one QALY.

One-way sensitivity analyses will also conducted for robustness: 1) Removing participants completing the survey within one year of diagnosis to remove any short-term treatment effects on HRQoL and costs within the first year of treatment, 2) Discount rates 3% and 7% 3) Willingness to pay for 1 QALY ($30,000, $70,000).

The economic evaluation report will follow the Consolidated Health Economic Evaluation Reporting Standards (CHEERS) statement^7^. All statistical and economic analyses were performed using R (ver. 4.4.1)(R Core Team 2023)^8^ packages Gmisc for plot and table output^9^, forestplot^10^, and knitr (Xie 2023)^11^ for reproducible research.

## Data flow chart

As for the IMPACT Study.

## Demographics

All the characteristics will be summarised by group. Where the total possible number of observations equals the applicable N_1_, N_2_,..., N as shown in the header row, the cell will be formatted n (%). Where the total possible number of observations is not equal to the applicable N_1_, N_2_, .., N as shown in the header row, the cell will be formatted n/N (%).

Table 2 Patient and clinical characteristics

|  | **Time to diagnosis** | |  | **Total N =** |  | **P value^**^** |
| --- | --- | --- | --- | --- | --- | --- |
|  | **<6 months N =** | **6+ months N =** |  |  |  |  |
| Age at symptom onset in years | Mean (SD) Median (IQR)  (missing=xx) |  |  | Mean (SD) Median (IQR)  (missing=xx) |  |  |
| Age at diagnosis in years | Mean (SD) Median (IQR)  (missing=xx) |  |  | Mean (SD) Median (IQR)  (missing=xx) |  |  |
| Age at survey in years | Mean (SD) Median (IQR)  (missing=xx) |  |  | Mean (SD) Median (IQR)  (missing=xx) |  |  |
| Time from diagnosis to completing survey in years (~time on treatment) | Mean (SD) Median (IQR)  (missing=xx) |  |  | Mean (SD) Median (IQR)  (missing=xx) |  |  |
| **Gender** | | | | | |  |
| Male | n (%) |  |  | n (%) |  |  |
| Female | n (%) |  |  | n (%) |  |  |
| **Ethnicity** | | | | | |  |
| White/European | n (%) |  |  | n (%) |  |  |
| Aboriginal Australian/Torres Strait Islander | n (%) |  |  | n (%) |  |  |
| Asian | n (%) |  |  | n (%) |  |  |
| Middle Eastern | n (%) |  |  | n (%) |  |  |
| Other | n (%) |  |  | n (%) |  |  |
| **State/Territory** | | | | | |  |
| ACT | n (%) |  |  | n (%) |  |  |
| NSW | n (%) |  |  | n (%) |  |  |
| QLD | n (%) |  |  | n (%) |  |  |
| SA | n (%) |  |  | n (%) |  |  |
| TAS | n (%) |  |  | n (%) |  |  |
| VIC | n (%) |  |  | n (%) |  |  |
| WA | n (%) |  |  | n (%) |  |  |
| **Parental education (highest level)** | | | | | |  |
| Did not complete high school | n (%) |  |  | n (%) |  |  |
| HSC or equivalent (Year 12) | n (%) |  |  | n (%) |  |  |
| College/TAFE/Diploma | n (%) |  |  | n (%) |  |  |
| University | n (%) |  |  | n (%) |  |  |
| Postgraduate | n (%) |  |  | n (%) |  |  |
| **^*^SEIFA Quintiles - IRSD** | | | | | |  |
| 1 - Most disadvantaged | n (%) |  |  | n (%) |  |  |
| 2 | n (%) |  |  | n (%) |  |  |
| 3 | n (%) |  |  | n (%) |  |  |
| 4 | n (%) |  |  | n (%) |  |  |
| 5 - Least disadvantaged | n (%) |  |  | n (%) |  |  |
| **^†^Remoteness** | | | | | |  |
| Major city | n (%) |  |  | n (%) |  |  |
| Inner regional | n (%) |  |  | n (%) |  |  |
| Outer regional | n (%) |  |  | n (%) |  |  |
| Remote | n (%) |  |  | n (%) |  |  |
| Very Remote | n (%) |  |  | n (%) |  |  |
| **Private health insurance** | | | | | |  |
| Yes | n (%) |  |  | n (%) |  |  |
| No | n (%) |  |  | n (%) |  |  |
| **Other clinical conditions** | | | | | |  |
| Uveitis | n (%) |  |  | n (%) |  |  |
| Psoriasis | n (%) |  |  | n (%) |  |  |
| Bone fractures | n (%) |  |  | n (%) |  |  |
| Dental problems | n (%) |  |  | n (%) |  |  |
| ^¶^Heart problems | n (%) |  |  | n (%) |  |  |
| Kidney damage | n (%) |  |  | n (%) |  |  |
| Diabetes | n (%) |  |  | n (%) |  |  |
| Other physical health problems | n (%) |  |  | n (%) |  |  |
| ^*^Socio-economic indices Australia, 2021, quintiles derived from national deciles by participant’s postal area, IRSD - Index of Relative Socio-economic Disadvantage  ^†^Remoteness measured by Accessibility/ Remoteness Index of Australia Plus (ARIA+) based on participant’s postal area  ^¶^For example, pericarditis, myocarditis, endocarditis, ventricular dysfunction  ^**^P values are chi-squared tests for proportions and t-tests for continuous variables | | | | | | |

## Costs

Table 3: Medications in the last year

| **Medications** | **Time to diagnosis** | | **Total**  **N** | **P value**** |
| --- | --- | --- | --- | --- |
|  | **<6 months N =** | **6+ months N =** |  |  |
| Methotrexate (Methoblastin, Trexject) | n (%) |  | n (%) |  |
| Hydroxychloroquine (Plaquenil) | n (%) |  | n (%) |  |
| Sulfasalazine (Salazopyrin, Pyralin) | n (%) |  | n (%) |  |
| Leflunomide (Arava, Arabloc) | n (%) |  | n (%) |  |
| Mycophenolate (Myfortic, Cellcept) | n (%) |  | n (%) |  |
| Adalimumab (Amgevita, Hadlima, Humira, Hyrimoz) | n (%) |  | n (%) |  |
| Etanercept (Brenzys, Enbrel, Frelzi) | n (%) |  | n (%) |  |
| Infliximab (Inflectra/Remsima Remicade, Renflexis) | n (%) |  | n (%) |  |
| Tocilizumab (Actemra) | n (%) |  | n (%) |  |
| Corticosteroids (oral) (Prednisone, Prednisolone, | n (%) |  | n (%) |  |
| Panafcortelone, Panafcort, Methylprednisolone) | n (%) |  | n (%) |  |
| Anti-inflammatories (Nurofen, Brufen, Mobic, Celebrex, Naprosyn, Orudis, Arcoxia, Voltaren, Feldene) | n (%) |  | n (%) |  |
| Corticosteroid INJECTIONS (into bone, joint or muscle) | n (%) |  | n (%) |  |
| How many in last six months? |  |  |  |  |
| 1-4 | n (%) |  | n (%) |  |
| 5-8 | n (%) |  | n (%) |  |
| 9-12 | n (%) |  | n (%) |  |
| 13-18 | n (%) |  | n (%) |  |
| 19-24 | n (%) |  | n (%) |  |
| >24 | n (%) |  | n (%) |  |
| Corticosteroid INFUSIONS (via a drip into the vein) | n (%) |  | n (%) |  |
| How many in last six months? |  |  |  |  |
| 1-4 | n (%) |  | n (%) |  |
| 5-8 | n (%) |  | n (%) |  |
| 9-12 | n (%) |  | n (%) |  |
| 13-18 | n (%) |  | n (%) |  |
| 19-24 | n (%) |  | n (%) |  |
| >24 | n (%) |  | n (%) |  |
| Other | n (%) |  | n (%) |  |

^**^P values are chi-squared tests for proportions and t-tests for continuous variables

Table 4: Medical investigations in last past year

| **Medical investigations** | **Time to diagnosis** | | **Total**  **N=** | **P value**** |
| --- | --- | --- | --- | --- |
|  | **<6 months N =** | **6+ months N =** |  |  |
| X-rays | Mean (SD)  Median (IQR)  (missing=xx) |  | Mean (SD)  Median (IQR)  (missing=xx) |  |
| Blood tests | Mean (SD)  Median (IQR)  (missing=xx) |  | Mean (SD)  Median (IQR)  (missing=xx) |  |
| MRI | Mean (SD)  Median (IQR)  (missing=xx) |  | Mean (SD)  Median (IQR)  (missing=xx) |  |
| Ultrasound | Mean (SD)  Median (IQR)  (missing=xx) |  | Mean (SD)  Median (IQR)  (missing=xx) |  |
| CT scan | Mean (SD)  Median (IQR)  (missing=xx) |  | Mean (SD)  Median (IQR)  (missing=xx) |  |
| Nuclear medicine scan | Mean (SD)  Median (IQR)  (missing=xx) |  | Mean (SD)  Median (IQR)  (missing=xx) |  |
| Eye examination | Mean (SD)  Median (IQR)  (missing=xx) |  | Mean (SD)  Median (IQR)  (missing=xx) |  |
| Other | Mean (SD)  Median (IQR)  (missing=xx) |  | Mean (SD)  Median (IQR)  (missing=xx) |  |

Table 5: Hospitalisation admissions in the last year by state

| **Hospitalisations** | **Time to diagnosis** | | **Total**  **N** | **P value**** |
| --- | --- | --- | --- | --- |
|  | **<6 months N =** | **6+ months N =** |  |  |
| Day Stay | n (%) |  | n (%) |  |
| Overnight | n (%) |  | n (%) |  |
| 2-4 nights | n (%) |  | n (%) |  |
| 5+ nights | n (%) |  | n (%) |  |
| **Reasons** | n (%) |  | n (%) |  |
| R1 | n (%) |  | n (%) |  |
| R2 | n (%) |  | n (%) |  |
| R3 | n (%) |  | n (%) |  |
| Other | n (%) |  | n (%) |  |

^**^P values are chi-squared tests for proportions and t-tests for continuous variables

Table 6: Medical professional visits in last year

| **Medical professional visits** | **Time to diagnosis** | |  |  |
| --- | --- | --- | --- | --- |
|  | **<6 months N =** | **6+ months N =** | **Total**  **N** | **P value**** |
| Primary physician | n (%) |  | n (%) |  |
| Paediatric rheumatologist | n (%) |  | n (%) |  |
| Adult rheumatologist | n (%) |  | n (%) |  |
| Orthopaedic specialist | n (%) |  | n (%) |  |
| Eye specialist (ophthalmologist) | n (%) |  | n (%) |  |
| Paediatrician | n (%) |  | n (%) |  |
| Psychiatrist | n (%) |  | n (%) |  |
| Dermatologist | n (%) |  | n (%) |  |
| Immunologist | n (%) |  | n (%) |  |
| Paediatric rheumatology nurse | n (%) |  | n (%) |  |
| Physiotherapist | n (%) |  | n (%) |  |
| Psychologist/Counsellor | n (%) |  | n (%) |  |
| Occupational therapist | n (%) |  | n (%) |  |
| Social worker | n (%) |  | n (%) |  |
| Dietitian/nutritionist | n (%) |  | n (%) |  |
| Podiatrist (feet) | n (%) |  | n (%) |  |
| Optometrist (eyes) | n (%) |  | n (%) |  |
| Exercise physiologist | n (%) |  | n (%) |  |
| Acupuncturist | n (%) |  | n (%) |  |
| Chiropractor/  Osteopath | n (%) |  | n (%) |  |
| Naturopath | n (%) |  | n (%) |  |
| Homeopath | n (%) |  | n (%) |  |
| Hydrotherapy | n (%) |  | n (%) |  |
| Other | n (%) |  | n (%) |  |
| None | n (%) |  | n (%) |  |

^**^P values are chi-squared tests for proportions and t-tests for continuous variables

Table 7: Annual government healthcare costs (medical investigations, health professional, hospital, and medications) related to JIA for the participants over the last 12 months

| Annual government healthcare costs | **Time to diagnosis** | |  |  | |  | **t-Test**  **P value** |
| --- | --- | --- | --- | --- | --- | --- | --- |
|  | **<6 months**  **Mean(SD) N =** | **6+ months**  **Mean(SD) N =** |  | **Total N =** | |  |  |
| **Total costs** | | | | | | | |
| ^*^Total government costs |  |  |  | |  |  |  |
| **Medical test costs** | | | | | | | |
| ^*^Total medical test costs |  |  |  | |  |  |  |
| **Health professional costs** | | | | | | | |
| ^*†^GPs |  |  |  | |  |  |  |
| ^*^Clinical specialists |  |  |  | |  |  |  |
| Rheumatology Nurse |  |  |  | |  |  |  |
| Allied Health |  |  |  | |  |  |  |
| Total health professional costs |  |  |  | |  |  |  |
| **Hospital costs** | | | | | | | |
| Mental health |  |  |  | |  |  |  |
| Injections/infusions |  |  |  | |  |  |  |
| Surgery |  |  |  | |  |  |  |
| Infection/virus |  |  |  | |  |  |  |
| Pain/inflammation/investigative |  |  |  | |  |  |  |
| Bone fracture |  |  |  | |  |  |  |
| Total hospital costs |  |  |  | |  |  |  |
| **Medication costs - PBS** | | | | | | | |
| ^§^csDMARDS |  |  |  | |  |  |  |
| ^¶^bDMARDS |  |  |  | |  |  |  |
| ^∥^tsDMARDS |  |  |  | |  |  |  |
| ^#^Corticosteriod ORAL |  |  |  | |  |  |  |
| ^**^All medications |  |  |  | |  |  |  |
| ^*^Applies the Medicare safety net as indicated by the participant ^†^Assumes participants visiting allied health completed a chronic disease care plan with GP ^‡^Assumes chronic disease plan rebates are claimed ^§^Assumes dosages: Methotrexate Oral and Subcutaneously 15mg per week (60% participants oral, 40% injections), Hydroxychloroquine 200mg daily, Suflasalazine 1000mg daily, Leflunomide 20mg daily ^¶^Assumes dosages: Adalimumab 40mg/0.8mL injection every 2 weeks, etanercept 50mg/mL injection weekly, Infliximab 120mg/mL injection monthly, Tocilizumab 162 mg/0.9 mL injection weekly, Abatacept 125 mg/mL injection weekly, Secukinumab 150 mg/mL injection weekly ^∥^Assumes dosages: Tofacitinib 5mg twice daily, Upadacitinib 15mg daily, Baricitinib 4mg daily ^#^Assumes dosages: Prednisone 25mg daily ^**^Excludes all other medications than DMARDS or ORAL corticosteriods. Note injections and infusions of corticosteriods are assumed to be included in hospitalisation costs | | | | | | | |

## Quality of Life

Child Quality of Life as measured by Child Health Utility (CHU9D) is summarised in Table 8 and Table 9.

Table 8 Child Quality of Life as measured by Child Health Utility (CHU9D)- Dimension scores

| **CHU9D dimension scores** | **<6 months**  **Mean (SD)**  **N =** | **6+ months**  **Mean (SD)**  **N =** |  |  | **t-Test**  **P value** |
| --- | --- | --- | --- | --- | --- |
| Worry |  |  |  |  |  |
| Sadness |  |  |  |  |  |
| Pain |  |  |  |  |  |
| Tiredness |  |  |  |  |  |
| Annoyance |  |  |  |  |  |
| School |  |  |  |  |  |
| Sleep |  |  |  |  |  |
| Daily routine |  |  |  |  |  |
| Activities |  |  |  |  |  |

Table 9 Child Quality of Life as measured by Child Health Utility (CHU9D) utility values

| **CHU9D Utility Values** | **Time to diagnosis** | | **Total**  **Mean (SD)**  **N=** | **t-Test**  **P value** |
| --- | --- | --- | --- | --- |
|  | **<6 months**  **Mean (SD)**  **N =** | **6+ months Mean (SD)**  **N =** |  |  |
| CHU9D utility value |  |  |  |  |

## Effectiveness measure

The effectiveness measure will be for aim 1: Incremental CHU9D Utility Value, and for aim 2. Incremental present value of Quality Adjusted Life Years, for the earlier time to diagnosis versus the later time to diagnosis.

Adjustments for imbalance in groups will be made if required.

Table 10 Effectiveness measures

| Effectiveness measures | **Time to diagnosis** | | **Mean difference**  **(95% CI)** | **Wald Test**  **P value** |
| --- | --- | --- | --- | --- |
|  | **<6 months N =**  **Coefficient**  **(95% CI)** | **6+ months N = Coefficient**  **(95% CI)** |  |  |
| Aim 1: CHU9D utility value |  |  |  |  |
| Aim 2: Present value^*^ of QALYs over lifetime horizon |  |  |  |  |

*Using life expectancies^3^, individual CHU9D utility values and 5% pa discount rate^4^

## Cost measure

The cost measure will be for aim 2: Incremental present value of costs for the earlier time to diagnosis versus later time to diagnosis. These will be the downstream health costs noting we are setting the intervention cost as zero. We are deriving the possible intervention cost that will still be cost effective.

Adjustments for imbalance in groups will be made if required.

Table 11 Cost measures

|  | **Time to diagnosis** | | **Mean difference**  **(95% CI)** | **Wald Test**  **P value** |
| --- | --- | --- | --- | --- |
|  | **<6 months N =**  **Coefficient**  **(95% CI)** | **6+ months N = Coefficient**  **(95% CI)** |  |  |
| Aim 2: Present value^*^ of costs over lifetime horizon |  |  |  |  |

*5% pa discount rate^4^

## Main outcomes

The main outcome will be the net benefit of diagnosis within 6 months using a willingnesss to pay (WTP) of $50,000 Australian dollars. An ICER of the Incremental cost per QALYs saved is unlikely to be appropriate given the intervention cost is set at zero and cost and QALY savings are anticipated. Table 12 presents incremental cost per QALYs saved net benefit instead of ICER and Figure 1 shows the presentation of a cost-effectiveness plane for the main outcome.

Table 12: Net benefit of diagnosis within 6 months

| **Analysis** | **Incremental costs in AUD (95% CI)** | **QALYs saved**  **Mean difference (95% CI)** | **Net benefit @ WTP of $50,000*** | **Bootstrapped Cost-Effectiveness plane estimates (% outcomes)** | | | |
| --- | --- | --- | --- | --- | --- | --- | --- |
|  |  |  |  | **NE** | **SE** | **SW** | **NW** |
| Diagnosis within 6 months versus 6+ months | $XX.XX  (XX.XX, XX.XX) | X.XX (X.XX, X.XX) |  |  |  |  |  |

95% CI, 95% Confidence Interval. WTP, Willingness to pay. Australian dollars

NW: More expensive and less effective

NE: More expensive and more effective

SW: Less expensive and less effective

SE: Less expensive and more effective

**Incremental cost in AUD**

**QALYs**

Figure 1 Cost-effectiveness plane for the main objective

## One way sensitivity analyses

One-way sensitivity analyses were also conducted for robustness: 1) Removing participants completing the survey within one year of diagnosis to remove any short-term treatment effects on HRQoL and costs within the first year of treatment, 2) Discount rates 3% and 7% 3) Willingness to pay for 1 QALY ($30,000, $70,000).

Results will be presented in a forest plot one for each of Incremental cost, incremental QALYs, and net benefit.

References

1. Stevens K. Developing a descriptive system for a new preference-based measure of health-related quality of life for children. *Quality of life research.* 2009;18(8):1105-1113.

2. Ratcliffe J, Huynh E, Chen G, et al. Valuing the Child Health Utility 9D: Using profile case best worst scaling methods to develop a new adolescent specific scoring algorithm. *Social science & medicine (1982).* 2016;157:48-59.

3. Australian Bureau of Statistics. Table 1.9, Life Tables, Life expectancy. ABS. <https://www.abs.gov.au/statistics/people/population/life-expectancy/2020-2022>. Published 2020-2022. Accessed 27 February, 2024, 2024.

4. NSW Treasury. NSW Government Guide to Cost-Benefit Analysis. In. Sydney (AU), 2023.

5. Australian Government Department of Health and Aged Care. Medicare Benefits Schedule Book, 1 November 2022. <https://www.mbsonline.gov.au/internet/mbsonline/publishing.nsf/Content/148049BCC6D57ED4CA25889F00110A8A/$File/1%20November%202022%20MBS%20Book.pdf>. Published 2022. Accessed 12 September, 2023, 2023.

6. Independent Hospital and Aged Care Pricing Authority. NWAU calculator for acute activity 2022-23. <https://www.ihacpa.gov.au/health-care/pricing/nwau-calculators>. Published 2022. Accessed 3 June, 2024, 2024.

7. Husereau D, Drummond M, Augustovski F, et al. Consolidated health economic evaluation reporting standards 2022 (CHEERS 2022) statement: updated reporting guidance for health economic evaluations. *International Journal of Technology Assessment in Health Care.* 2022;38(1):e13.

8. R Core Team. R: A Language and Environment for Statistical Computing. R Foundation for Statistical Computing. <https://www.R-project.org/>. Published 2024. Accessed.

9. Gordon M. Gmisc: Descriptive Statistics, Transition Plots, and More. R package version 3.0.3 Web site. <https://CRAN.R-project.org/package=Gmisc> Published 2023. Accessed.

10. M Gordon TL. forestplot: Advanced Forest Plot Using 'grid'. R package version 3.1.3 Web site. <https://gforge.se/packages/>. Published 2023. Accessed.

11. Xie Y. knitr: A General-Purpose Package for Dynamic Report Generation in R. R package version 1.45 Web site. <https://yihui.org/knitr/>. Published 2023. Accessed.
